# How Do Older People Experience Hospital to Home Discharge? An Overview of Qualitative Reviews

**DOI:** 10.1111/opn.70061

**Published:** 2026-02-18

**Authors:** Úna Kerin, Judith Dyson, Fiona Cowdell

**Affiliations:** ^1^ School of Nursing and Midwifery Birmingham City University Birmingham UK

**Keywords:** hospital discharge, older people, overview review, qualitative

## Abstract

**Introduction:**

Prolonged hospital stays can increase the risk of hospital‐acquired adverse events among older people, and this can give rise to increasingly complex care needs following discharge from hospital. The unique experiences of older people are important to inform effective healthcare service design. This review aims to better understand our current knowledge regarding the experiences of older people during hospital to home discharge through examining (i) the characteristics of older people included in research regarding hospital to home discharge and (ii) older people's experiences of hospital to home discharge.

**Methods:**

An overview of qualitative reviews methodology was applied. CINAHL, MEDLINE, PsycINFO and the Cochrane Library databases were systematically searched from inception to October 2024, using a combination of keywords and database specific terms and reporting followed PRISMA 2020 guidance. To estimate the extent of overlap among primary studies included in the reviews, the formula for calculated covered area (CCA) was applied. Data were extracted and analysed according to the aims of this review, and results were thematically synthesised.

**Results:**

Six qualitative reviews reporting on 98 international primary research studies were included. Analysis revealed mixed and somewhat limited reporting of older person characteristics including age, gender, ethnicity, health conditions and reasons for hospital admission. All studies offered some insight into older persons' experiences of hospital discharge; half included the views of lay carers and healthcare practitioners. Five core themes were derived from inductive analysis: (i) wanting to be at home, (ii) working together and communication, (iii) the system versus the person, (iv) failing to meet needs and (v) role of family carers. The overarching finding was older people want to be at home but feel uninvolved in planning and therefore poorly prepared for discharge.

**Conclusion:**

This review exposes limited research addressing the older persons' gender, ethnicity, existing health conditions and reason for admission suggesting gaps in our understandings of the older person and their unique home context in existing research. More detailed reporting of older persons' individual characteristics and greater attention to direct reports from older persons would enrich our understanding of older persons' unique hospital to home discharge experience in different contexts. This more detailed understanding might serve to advance more bespoke strategies to enhance person‐centred discharge processes and inform future research.

**Implications for Practice:**

In communicating, healthcare practitioners need to be actively ‘present’ to enable older people to effectively engage in discharge planning, enhance autonomy and promote shared decision making.

**Summary**

**What does this research add to existing knowledge in gerontology?**

- This review exposes limited research addressing older persons' gender, ethnicity, existing health conditions and reason for admission, suggesting gaps in our understandings of the older person and their unique home context in existing research regarding hospital home discharge.
- Many older people are aware of being in a ‘busy’ and ‘rushed’ healthcare environment and this can impact communication and active engagement in discharge planning.

**What are the implications of this new knowledge for nursing care for and with older adults?**

- In communicating, all healthcare practitioners need to be actively ‘present’ to enable more effective interactions, enhance autonomy and promote shared decision making.

**How could the findings be used to influence practice, education, research, and policy?**

- To avoid homogenisation and to support greater diversity and inclusivity of older people, researchers should provide information about age, gender, ethnicity, health conditions and reasons for admission. Further research with diverse groups of older people is needed to understand unique barriers and facilitators that allow healthcare leaders and policymakers to plan and implement an acceptable and safe person‐centred hospital to home discharge.

## 1 Introduction

Globally, the number of people aged ≥ 60 years is expected to increase from 12% to 22% by 2050 (World Health Organisation 2024). The number of older people living with four or more health conditions is expected to double by 2035 (Kingston et al. 2018). These predicted figures are likely to increase more rapidly with recent research suggesting that almost a third of people aged ≥ 65 years who acquired COVID‐19 during the pandemic have gone on to develop one or more new clinical conditions (Cohen et al. 2022). People living with multiple health conditions account for the most hospital admissions, longer hospital stays (NHS Digital 2024) and a high proportion of healthcare spending (Tanke et al. 2019). Protracted hospital stays contribute to disease burden for older people (Facchinetti et al. 2021) by increasing the risk of hospital‐acquired adverse events including healthcare‐associated infection (Boncea et al. 2021), sarcopenia (Welch et al. 2018), worsening nutritional status (Roberts et al. 2019), falls, clinical deterioration, pressure ulcers and reduced wound healing (Dent et al. 2019). Prolonged hospitalisation is also associated with feelings of powerlessness (van der Meide et al. 2015), boredom, frustration and low mood (Clarke et al. 2018). Even after a short hospital admission, frail older people have an increased risk of mortality after discharge home (Keeble et al. 2019).

Considering the significant impact of hospitalisation on older people and the potential for increasingly complex care needs following discharge from hospital (Kuluski et al. 2017), it is unsurprising that international healthcare leaders and policymakers are interested in exploring interventions to minimise the duration of hospitalisation, best practice in discharge planning (Zurlo and Zuliani 2018) and reduce hospital readmissions for older people (Coffey et al. 2017). This is accompanied by growing evidence which suggests that many older people prefer to receive care in their own home (Kasper et al. 2019). However, facilitating community‐based home care is challenging health systems around the world. For example, in the United Kingdom, post‐COVID‐19 healthcare service demand currently outstrips National Health Service (NHS) resource availability. For context, in 2022, there were over 6 million people on NHS waiting lists for pre‐planned care (Stoye et al. 2022). This is accompanied by acute and community hospital bed shortages partially owing to delayed discharges which are often attributable to acute/community staff being unable to arrange sufficient social support services to enable a safe discharge (Plewes 2022). This example illustrates a ‘mismatch’ between demand for community care support and healthcare provider capacity to deliver these services (Gridley et al. 2022).

This ‘mismatch’ between service demand and capacity to deliver care services in the community is likely to impact older people who often have complex discharge needs (Liljas et al. 2022). Although this evidence is from the United Kingdom, the challenges of providing post hospital discharge care which meets individual needs are replicated internationally (WHO 2024). Enabling a successful person‐centred hospital to home discharge for older people is vital to addressing one facet of this complex wider issue. Therefore, it is timely to provide health professionals with a comprehensive and contemporary overview of reviews reporting older people's experiences of hospital to home discharge. Considering that several literature reviews exist on this topic, an overview of qualitative reviews approach was employed as it is an effective method of comparing multiple reviews and summarising review evidence (Hunt et al. 2018; Pollock et al. 2016). Through this overview of reviews, we can better understand our current knowledge regarding (i) the characteristics of older people who have been included in research regarding hospital to home discharge and (ii) older people's experiences of hospital to home discharge.

## 2 Method

Overviews of qualitative reviews are often referred to as umbrella reviews or a review of reviews, and this methodological process offers a comprehensive synthesis regarding a specific subject (Aromataris et al. 2015; Pollock et al. 2016). The review comprised the following steps: (i) development of a priori protocol, (ii) pre‐specified review question with clear inclusion and exclusion criteria, search strategy and data extraction and quality appraisal methods and (iii) reporting of defined and repeatable methods of data synthesis. This review has been reported using the Preferred Reporting Items for Systematic Reviews and Meta‐analyses (Page et al. 2021).

### 2.1 Pre‐Specified Review Question

How do older people experience hospital to home discharge?

Objective 1: Map out the characteristics of older people included in research regarding hospital to home discharge.

Objective 2: Synthesise knowledge regarding older people's experiences of hospital to home discharge.

### 2.2 Inclusion and Exclusion Criteria

Inclusion criteria were (i) all qualitative literature review methods, (ii) that examined discharge of older people from hospital to home, (iii) considered the older person's thoughts, feelings and experiences, (iv) it was possible to disaggregate the older person's views and (v) published in English language. Exclusion criteria comprised (i) focus on end‐of‐life hospital to home discharge and the discharge or transfer of care from hospital to care home, nursing home, hospice or rehabilitation facility and (ii) evaluating the outcomes or engagement with specific transitional care interventions used across care settings.

### 2.3 Search Strategy and Selection Process

**FIGURE 1:**
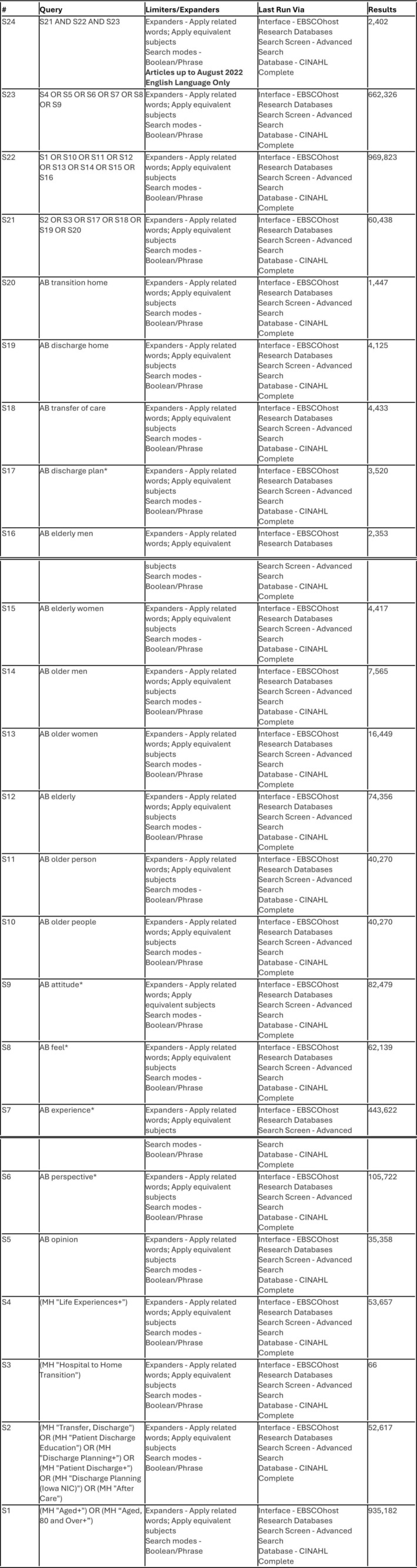
Example search strategy.

**FIGURE 2:**
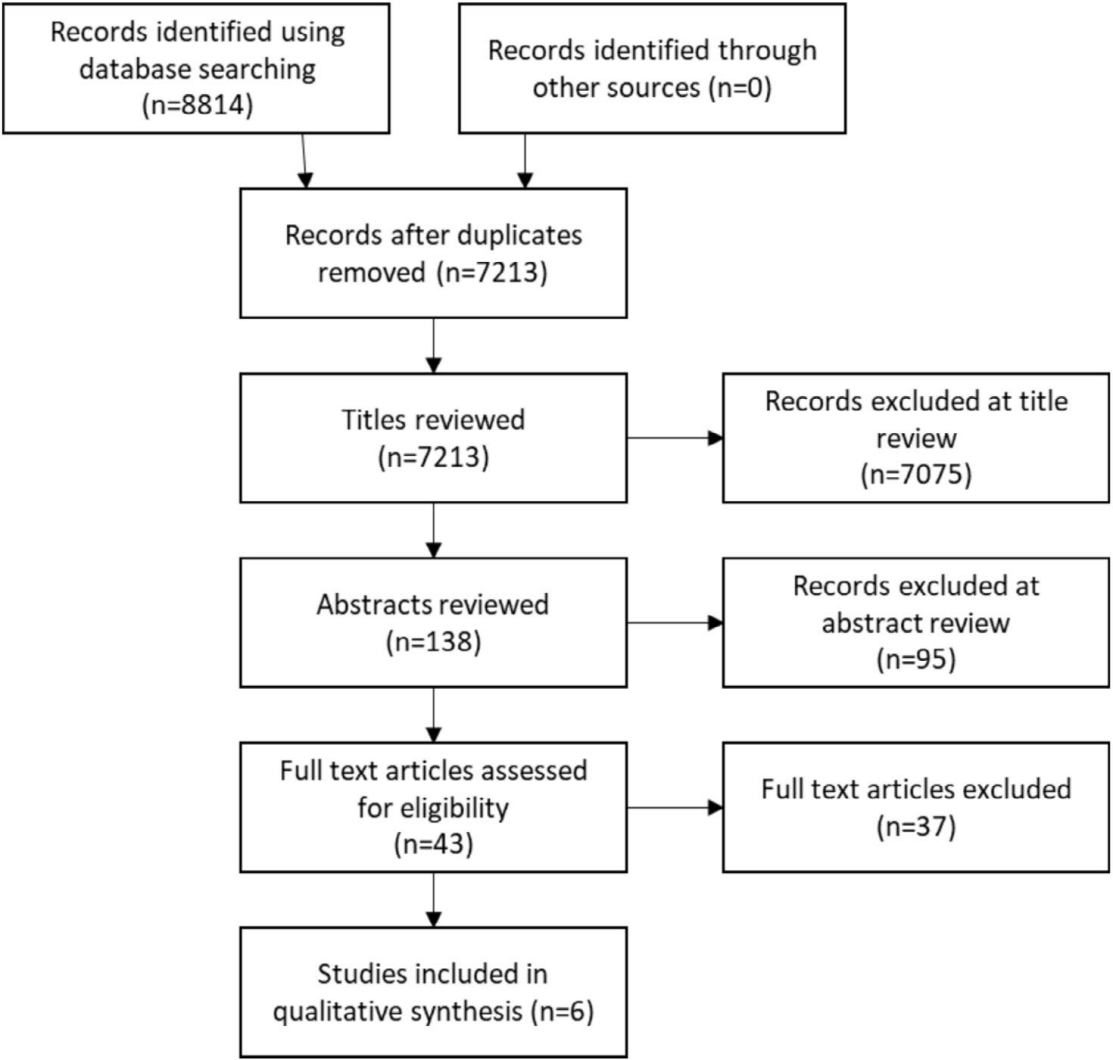
PRISMA flowchart.

A comprehensive systematic healthcare database search, designed with a subject specialist academic librarian, included Cumulated Index to Nursing and Allied Health Literature (CINAHL), Medical Literature Analysis and Retrieval System Online (MEDLINE), PsycINFO and the Cochrane library, from inception to October 2024. Keywords and database‐specific Main Subject Headings (MeSH) were derived from pre‐determined population, experience and outcomes (PEO) (Bettany‐Saltikov and McSherry 2024). An example search strategy from CINAHL is presented in Figure 1; our strategy had to be adapted for each database. A PRISMA flowchart (Page et al. 2021) outlining the article screening and selection process is presented in Figure 2. The process involved de‐duplication, screening title and abstract and full review of remaining papers. The selection was completed by two authors with discussion with the third to resolve any disagreements. Excluded papers did not focus on older people, home discharge, or it was not possible to disaggregate older person data. Others were excluded as the emphasis was on interventions.

### 2.4 Data Extraction

Data extraction focused on Objective 1, *which* older people have been included in research regarding hospital to home discharge. We therefore extracted data regarding aim, review design and date range, number and country of articles and older person participant demographics comprising age, gender, ethnicity, health conditions and reason for admission. Phase II focused on data extraction relating to patient experiences. This process was conducted by one author (UK) and authors JD and FC then reviewed.

### 2.5 Quality Appraisal

Considering the differing review approaches and to allow some comparison between reviews, we used the 10‐point Critical Appraisal Skills Programme (CASP 2024) systematic review checklist and adapted it to better reflect quality standards in qualitative reviews. Due to the limited number of available reviews, we chose not to exclude based on score. We adapted two questions to ensure relevance to our review. Question six was changed from ‘What are the overall results of the review?’ to ‘are the results of the study clear?’ and question seven from ‘How precise are the results?’ to ‘are the results justified?’ Each question can be answered yes, cannot tell or no. Final scores were checked and agreed by all authors. Scores ranged from 8/10 to 10/10. No questions were answered ‘no’. Reasons for ‘cannot tell’ responses were lack of a clearly focused review question (*n* = 1), questionable applicability to the local population (*n* = 2) and no reported consideration of benefits or harms (*n* = 2).

### 2.6 Data Synthesis

To estimate the extent of overlap among primary studies included in the reviews, the formula for calculated covered area (CCA) was applied as per Pieper et al. (2014). Data relating to patient experiences were thematically analysed according to a six stage inductive process (familiarisation, coding, initial theming, review themes, define themes and report) (Braun and Clarke 2006). All authors familiarised themselves with the papers (*n* = 6); initial coding and theming was completed by UK, and all authors then reviewed and defined themes in preparation for reporting.

## 3 Results

A summary of included reviews, detailing *which* older people have been included in research relating to discharge planning is presented in Table 1 and a summary of older people's experiences with resulting themes is presented in Table 2. The CASP (2024) quality appraisal score of each article is shown in Table 3. The characteristics and attributes of included papers and participant demographics are reported alongside the five main themes below.

**TABLE 1:** Summary of included reviews.

First author, year	Aim	Review design and search range	Number of articles and countries represented	Older person demographics
Allen (2017)	Understand older people, carers and health providers experiences of transition from hospital to home	Meta‐synthesis 1990–2014	Total = 20. Australia (*n* = 3), Canada (*n* = 3), Sweden (*n* = 2), UK (*n* = 3), USA (*n* = 8), Ireland (*n* = 1)	Age: ≥ 60 years, Gender: not reported, Ethnicity: not reported, Health conditions: long ‐term conditions, Reason for admission: not reported
Hestevik (2019)	Summarise older people's experiences of adapting to life at home after discharge	Qualitative meta‐summary 2006–2017	Total = 13. Denmark (*n* = 1), unknown (*n* = 1), Australia (*n* = 3), USA (*n* = 2), Sweden (*n* = 2), UK (*n* = 1), Ireland (*n* = 1), Canada (*n* = 1), New Zealand (*n* = 1)	Age: ≥ 65 years (not reported 2/13 papers), Gender: men and women, Ethnicity: not reported, Health conditions: various, Reason for admission: various (reported in 10/13 papers)
Lilleheie (2019)	Report experiences of older people's participation in hospital discharge	Qualitative meta‐summary 1994–2017	Total = 18. Canada (*n* = 4), Sweden (*n* = 5), Norway (*n* = 3), UK (*n* = 3), New Zealand (*n* = 1), USA (*n* = 2)	Age: ≥ 65 years (not reported in 2/18 papers) Gender: men and women, Ethnicity: not reported Health conditions: various, Reason for admission: various (reported 9/18 papers)
Murray (2019)	Develop a model of patient‐enacted involvement in care with older people and informal carers	Systematic review 2005–2019	Total = 16. Canada (*n* = 2), USA (*n* = 1), Italy (*n* = 1), Ireland (*n* = 1), Sweden (*n* = 2), UK (*n* = 4), Norway (*n* = 2), Denmark (*n* = 2), Australia (*n* = 1)	Mean age: ≥ 59, Gender: men and women, Ethnicity: reported in 3/16 papers, Health conditions: various (reported in 11/16 papers), Reason for admission: various (reported in 11/16 papers)
Ozavci (2021)	Explore experiences of managing medications post‐discharge with older people, informal carers and healthcare practitioners	Systematic review 1979–2019	Total = 33. Australia (*n* = 4), Hong Kong (*n* = 1), USA (*n* = 12), UK (*n* = 5), Canada (*n* = 4), Sweden (*n* = 1), New Zealand (*n* = 1), Denmark (*n* = 1), Italy (*n* = 2), Norway (*n* = 1), Belgium (*n* = 1)	Age: not reported in 30/33 papers, Gender: men and women, Ethnicity: not reported, Health conditions: multiple (reported in 1/33 paper), Reason for admission: not reported
Perry (2012)	Explore older people's experience of discharge from hospital following orthopaedic surgery	Meta‐synthesis 1950–2010	Total = 16. UK (*n* = 2), Japan (*n* = 1), Australia (*n* = 2), Sweden (*n* = 4), Canada (*n* = 2), New Zealand (*n* = 1), Finland (*n* = 1), USA (*n* = 3)	Mean or median age: < 60 years, Gender: men and women, Ethnicity: reported in 4/16 papers, Health conditions: multiple (reported in 1/16 paper), Reason for admission: orthopaedic surgery

**TABLE 2:** Summary of themes.

Author and date	Theme				
	Wanting to be at home	Working together and communication	System vs. the person	Failing to meet needs	Role of family carers
Allen (2017)	Patients wanted to return to their own homes but experienced multiple issues post‐discharge	Inconsistencies in care coordination among and between providers, service users and carers. Lack of clarity about roles and responsibilities		Patients and carers experienced unmet needs post‐discharge including support with symptom management, help with activities of living and psychosocial support	Patients often need support from family members to help them adjust to returning home. This could change the nature of relationships
Hestevik (2019)	Most wanted to be at home but struggled to cope with activities of daily living leading and physical condition and restrictions led to missed social interaction, loneliness and depression	Patients felt unseen, unheard, excluded, ‘patronised’. Care could be paternalistic, patients tended to accept discharge plans without question. Staff often ‘distant’ and ‘stressed’	Rushed and poorly planned. Not tailored to individual need. Staff stressed, distant, hurried, some people felt ‘objectified’	Patients often felt ‘insecure’ and ‘unsafe’ on discharge due to lack of information about diagnosis, care and management	Patients benefited from strong relationships with family and friends but did not want to burden them with meeting their needs that could lead to feelings of anxiety and guilt
Lilleheie (2019)	Feelings of helplessness. Nearly all older people wanted to go home but some were concerned about their ability to manage	Patients could struggle to understand and remember information Some reported one‐way communication, jargon, powerlessness, resignation and did not feel equal participants in the discharge process	Discharge driven by speed and cost, clearing hospital beds and felt ‘standardised’ and pressured	Limited engagement in the discharge process was a stressor for some patients	Older people used their families to support their inclusion and overcome barriers to communication and information sharing
Murray (2019)		Patients respond to interactions with HCPs with varying degrees of involvement including ‘non‐involvement’, ‘information‐acting’, ‘challenging and chasing’ and ‘autonomous‐acting’. Some perceived exclusion and staff appeared ‘unapproachable’ and ‘authoritarian’	Staff appeared busy and focused on the discharge process rather than individual's needs and preference		
Ozavci et al. (2021)	Older people wanted post‐discharge support at home	Older people frequently reported ineffective communication. Many were not confident to ask questions	Older people wanted discharge plans tailored to their individual circumstances and needs	Patients reported insufficient knowledge about medication regimens	Uncertainty about medications increased reliance on family members
Perry et al. (2012)	Patients reported needing determination and drive to get home. Most experience increased level of need in the short‐term	Good communication and information enhanced patient confidence			Family crucial to recovery, adaptation, accepting help could be difficult

**TABLE 3:** CASP quality appraisal.

Question	Allen (2017)	Hestevik (2019)	Lilleheie (2019)	Murray (2019)	Ozavci (2021)	Perry (2012)
1. Did the review address a clearly focused question?	Y	Y	Y	NR	Y	Y
2. Did the authors look for the right type of papers?	Y	Y	Y	Y	Y	Y
3. Do you think all the important, relevant studies were included?	Y	Y	Y	Y	Y	Y
4. Did the review's authors do enough to assess quality of the included studies?	Y	Y	Y	Y	Y	Y
5. If the results of the review have been combined, was it reasonable to do so?	Y	Y	Y	Y	Y	Y
6. What are the overall results of the review? (are the results of the study clear?)	Y	Y	Y	Y	Y	Y
7. How precise are the results? (are the results justified?)	Y	Y	Y	Y	Y	Y
8. Can the results be applied to the local population?	NR	Y	Y	NR	Y	Y
9. Were all important outcomes considered?	Y	Y	Y	Y	Y	Y
10. Are the benefits worth the harms and costs?	NR	Y	Y	Y	NR	Y
Total score	8/10	10/10	10/10	8/10	9/10	10/10

Abbreviations: NR, not reported; Y, yes.

### 3.1 Characteristics and Aims of Reviews

Six reviews reporting on 98 studies were included. These reviews were published between 2012 and 2021. The latest review identified in our search to October 2024 was Ozavci et al. (2021) with 33 studies included. To estimate the extent of overlap among primary studies included in the reviews, the formula for calculated covered area (CCA) was applied (Pieper et al. 2014). A CCA of 2.8% indicates ‘slight overlap’ (Pieper et al. 2014). Only 12 of the 100 studies were included in more than one review (see Table 4). Despite the varied focus and lens of all six reviews, they all considered the older person's perspective of hospital to home discharge. Perry et al. (2012) focused on discharge following orthopaedic surgery. Two reviews explored the experiences involved in discharge planning (Lilleheie et al. 2019; Murray et al. 2019), one focused on understanding care integration (Allen et al. 2017), one explored adaptation to post‐discharge life (Hestevik et al. 2019), and one examined experiences of managing medication (Ozavci et al. 2021). All reviews were led by experts on quality and safety (Murray et al. 2019; Ozavci et al. 2021), physiotherapy (Hestevik et al. 2019; Lilleheie et al. 2019; Perry et al. 2012) and nursing (Allen et al. 2017). Three reviews sought the views of only older people (Lilleheie et al. 2019; Hestevik et al. 2019; Perry et al. 2012), one included older people and informal carers (Murray et al. 2019), and two included older people, informal carers and healthcare practitioners (HCPs) (Allen et al. 2017; Ozavci et al. 2021).

**TABLE 4:** Primary studies overlap.

Primary studies	Literature reviews					
	Allen (2017)	Hestevik (2019)	Murray (2019)	Ozavci (2021)	Lilleheie (2019)	Perry (2012)
Allen et al. (2018)				X		
Andreasen et al. (2015)		X	X			
Archibald (2003)						X
Armitage and Kavanagh (1995)	X					
Armitage and Kavanagh (1996)	X					
Arora et al. (2010)				X		
Bagge et al. (2014)		X	X	X		
Barnett et al. (2017)				X		
Bayliss et al. (2016)				X		
Blennerhassett and Hilberts (2011)				X		
Bull and Roberts (2001)	X					
Bull (1992)	X					
Bull (1994)	X				X	
Burns et al. (1992)				X		
Byrne et al. (2011)	X		X			
Chapin et al. (2014)	X					
Chiu et al. (2018)				X		
Clare et al. (1998)				X		
Cochrane et al. (1992)				X		
Coleman et al. (2004)				X		
Coleman et al. (2002)	X					
Dedhia et al. (2009)				X		
Del Sindaco et al. (2007)				X		
Dilworth et al. (2012)		X				
Dossa et al. (2012)		X	X			
Durocher et al. (2015)					X	
Durocher et al. (2017a)					X	
Durocher et al. (2017b)					X	
Dyrstad et al. (2015)					X	
Efraimsson et al. (2006)					X	
Ekdahl et al. (2012)					X	
Ellins and Glasby (2014)			X			
Enguidanos and Brumley (2016)				X		
Eyler et al. (2016)				X		
Flacker et al. (2007)				X		
Foust et al. (2012)	X					
Fujita et al. (2006)						X
Gabrielsson‐Jarhult & Nilsen (2016)					X	
Gadbois et al. (2019)				X		
Georgiadis and Corrigan (2017)			X			
German et al. (1982)				X		
Giosa et al. (2014)			X			
Graham et al. (2009)	X					
Grant et al. (2009)						X
Grimmer et al. (2004)	X					
Gustafsson et al. (2007)						X
Gustafsson et al. (2010)						X
Harvey et al. (2017)			X			
Heine et al. (2004)						X
Huby et al. (2007)	X					
Hvalvik and Dale (2015)			X			
Hvalvik and Reierson (2015)			X			
Hvidt et al. (2014)				X		
Jeffs et al. (2017)				X		
Jones (2012)		X				
Karlsson et al. (2016)		X				
Knight et al. (2011)		X		X	X	
Knight et al. (2013)			X			
Laugaland et al. (2014)					X	
LeClerc et al. (2002)	X					
Leduc et al. (1998)				X		
Lindquist et al. (2014)				X		
Loft et al. (2003)						X
Lopez Cabezas et al. (2006)				X		
Marcinkowski et al. (2005)						X
McAiney et al. (2017)				X		
McBride (1994)					X	
McKeown (2007)		X	X			
McWilliam and Sangster (1994)	X					
McWilliam (1992)	X					
Mesteig et al. (2010)				X		
Montin et al. (2002)						X
Neiterman et al. (2015)		X	X			
Nyborg et al. (2017)					X	
O'Kula et al. (2016)				X		
Perry et al. (2012)		X			X	
Plank et al. (2012)			X			
Popejoy (2011)					X	
Procter et al. (2001)	X					
Rastogi et al. (2007)						X
Reay et al. (2015)		X				
Rich et al. (1996)				X		
Robinson (1999)						X
Rustad et al. (2016)			X	X		
Rydeman and Törnkvist (2006)	X					
Rydeman and Törnkvist (2010)	X	X	X		X	
Sexton and Brown (1999)				X		
Shen et al. (2006)				X		
Showalter et al. (2000)						X
Slayter et al. (2013)		X				
Spinewine et al. (2007)				X		
Swinkels and Mitchell (2009)			X		X	
Williams et al. (1994)						X
Wong and Hogan. (2016)				X	X	
Woolhead et al. (2005)						X
Zakrajsek et al. (2013)	X					
Ziden et al. (2008)						X
Ziden et al. (2010)						X

*Note:* The blue highlighted sections indicate those articles which overlap.

### 3.2 Search, Review and Analysis Methods

Cumulatively, reviews searched for published studies from 1950 to 2019. Databases included CINAHL, Embase, MEDLINE, PsycINFO, Google Scholar, INFORMIT, Masterfile Premier, Socindex, Cochrane library, Joanna Briggs Institute, Allied and Complementary Medicine (AMED), Ovid Nursing Database, Web of Science, Proquest and Academic Search Complete or Premier. Review methods included systematic review (*n* = 2), meta‐synthesis (*n* = 2) and meta‐summaries (*n* = 2). Data analysis methods included thematic analysis (*n* = 2), qualitative meta‐summary (*n* = 2), a framework approach (*n* = 1) and a deductive and inductive analysis (*n* = 1).

### 3.3 Quality Appraisal

The six reviews assessed quality using: Joanna Briggs Assessment and Review Instrument (*n* = 2), Critical Appraisal Skills Programme (*n* = 2), Mixed Methods Appraisal Tool (*n* = 1) and an adapted version of Consolidated Criteria for Reporting Qualitative Research (*n* = 1).

### 3.4 Countries

**FIGURE 3:**
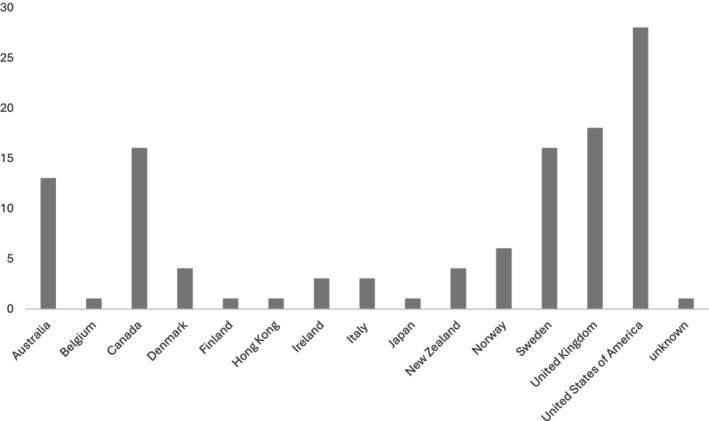
Summary of countries included in reviews.

The reviews were conducted by authors in Australia (Allen et al. 2017), Norway (Hestevik et al. 2019; Lilleheie et al. 2019), the United Kingdom (Murray et al. 2019; Ozavci et al. 2021) and New Zealand (Perry et al. 2012). All reviews reported countries represented, these included Canada, United Kingdom, United States, Australia, Sweden, New Zealand, Norway, Ireland, Denmark, Italy, Finland, Japan, Hong Kong and Belgium, numbers of papers are summarised in Figure 3.

### 3.5 Participant Demographics

Older age was an inclusion criterion for all papers. Three reviews reported including people aged ≥ 65 years (Lilleheie et al. 2019; Hestevik et al. 2019; Ozavci et al. 2021), two those aged ≥ 60 years (Allen et al. 2017; Perry et al. 2012) and one people with a mean age of ≥ 60 years (Murray et al. 2019). However, there were deficits in reporting, for example, two of 18 studies (Lilleheie et al. 2019), two of 13 (Hestevik et al. 2019), one of 16 (Murray et al. 2019) and 30 of 33 (Ozavci et al. 2021) did not report age. Gender is not reported in two reviews (Allen et al. 2017; Ozavci et al. 2021), and ethnicity is not reported in four (Allen et al. 2017; Lilleheie et al. 2019; Hestevik et al. 2019). Details on health conditions and reasons for admission are generic, except for Perry et al. (2012) who focused on people undergoing orthopaedic surgery.

### 3.6 Thematic Analysis of Older Peoples' Experiences of Hospital to Home Discharge

The overarching finding was older people want to be at home but feel uninvolved in planning and therefore poorly prepared for discharge. Themes from included reviews are summarised in Table 2. Our inductive synthesis of reviews identified five themes: (i) wanting to be at home, (ii) working together and communication, (iii) the system versus the person, (iv) failing to meet needs and (v) role of family carers. Each theme is presented in turn below.

#### 3.6.1 Theme 1: Wanting to be at home

The wish to be at home was virtually universal; however, this was tinged with fear (Murray et al. 2019) and concerns about ability to manage (Lilleheie et al. 2019; Hestevik et al. 2019). Mental outlook, including encompassing the notions of faith, confidence, appreciation, and motivation, was a key factor influencing successful discharge (Perry et al. 2012). Lack of control over the discharge process exacerbated concerns, and people reported wanting to be sure that they were ready and that the necessary discharge support would be in place (Ozavci et al. 2021). Information on how to manage at home increased confidence (Perry et al. 2012).

#### 3.6.2 Theme 2: Working together and communication

Limited communication was reported in all but one (Perry et al. 2012) review. This included not being listened to, with some people feeling patronised (Hestevik et al. 2019). The use of jargon made it difficult for patients to fully engage (Lilleheie et al. 2019). Multiple people being involved in the process made it unclear who was responsible for what (Allen et al. 2017) and hindered effective communication (Allen et al. 2017; Hestevik et al. 2019). Patients suggested asymmetric relationships with the balance of power being weighted towards staff (Lilleheie et al. 2019). Levels of engagement and autonomy varied from non‐involvement to ‘autonomous acting’ (Murray et al. 2019). It appeared that some who may have wanted autonomy became resigned to being passive in the process (Murray et al. 2019). People wanted to participate authentically (Lilleheie et al. 2019; Hestevik et al. 2019) but often found this impossible to achieve. Even when individual staff were working alongside patients, it was reported that the system was not designed to meet person‐centred needs.

#### 3.6.3 Theme 3: The System Versus the Person

Older people were acutely aware of healthcare system and staffing pressures (Lilleheie et al. 2019; Hestevik et al. 2019; Murray et al. 2019; Ozavci et al. 2021). Reports of ‘speed’, ‘rush’, ‘busy’, ‘pressured’ and ‘hurried’ were frequent; these were a hindrance and made older people reluctant to engage (Lilleheie et al. 2019; Hestevik et al. 2019; Murray et al. 2019). Many reported experiences of discharge planning lacking focus on their particular needs and feeling standardised (Lilleheie et al. 2019; Murray et al. 2019; Ozavci et al. 2021). At the extreme people felt ‘objectified’ with no consideration of them as a human being with individual needs (Hestevik et al. 2019).

#### 3.6.4 Theme 4: Failing to Meet Needs

Failure to meet all the older person's perceived needs was identified in three reviews (Allen et al. 2017; Lilleheie et al. 2019; Hestevik et al. 2019). People reported having to adjust in the face of poor discharge planning and limited provision of services on return home (Allen et al. 2017). Lack of advice regarding symptom management was problematic (Allen et al. 2017). People interviewed post‐discharge reported lack of information about diagnosis, future care and they struggled to cope with day‐to‐day life, and their physical condition and restrictions led to social isolation and low mood (Hestevik et al. 2019). Even when information had been given, older people reported struggling to understand and remember (Lilleheie et al. 2019). People considered there was inadequate preparation in terms of being able to meet their activities of daily living or psychosocial preparedness (Allen et al. 2017). Suboptimal discharges led to the need for significant reliance on help from family carers.

#### 3.6.5 Theme 5: Role of Family Carers

Four papers reported the crucial nature of the involvement of family carers (Allen et al. 2017; Lilleheie et al. 2019; Hestevik et al. 2019; Perry et al. 2012). In the discharge process, older people used their families to enable their inclusion (Lilleheie et al. 2019). Post‐discharge, patients became acutely aware of their increased reliance on help from family members when more assistance was needed (Hestevik et al. 2019). This was problematic for some. Reduced independence was hard to live with (Perry et al. 2012). Changes in the nature of the relationship were of concern to some older people both during the discharge process and afterwards (Allen et al. 2017). Although they recognised the need for help, some people found adapting and accepting this difficult (Perry et al. 2012).

## 4 Discussion

To the best of our knowledge, this is the first overview of qualitative reviews examining how older people experience hospital to home discharge alongside the characteristics of the older people included in this research. Collectively, the six reviews reported on 98 primary studies published between 2012 and 2021 and included international research from a range of developed countries with the United States, the United Kingdom, Sweden, Canada and Australia dominating the field. In general, people aged ≥ 65 years were categorised as older. Data about the older persons' gender, ethnicity, existing health conditions and reasons for hospitalisation is limited. It is possible that this was reported in primary studies but not included in the reviews, but absence here leads to a homogenisation of older people and limits understanding of the nuances of person and context‐specific discharge.

Reviews took differing perspectives on discharge experiences; however, collectively, five themes were derived from inductive analysis: (i) wanting to be at home, (ii) working together and communication, (iii) the system versus the person, (iv) failing to meet needs and (v) role of family carers. The overarching finding was older people want to be at home but felt uninvolved in planning and therefore poorly prepared for discharge. Lack of involvement had many causes but was often influenced by poor communication and older people's awareness of systemic pressures. Person‐centred needs were often not met, leading to some experiencing feelings of insecurity or vulnerability. Reliance on help from family and friends often escalated following discharge home and could be challenging in terms of finding it difficult to accept help, not wanting to be a burden and changed relationships.

Our review demonstrates that despite current policy and rhetoric there remains a need to improve the person‐centred nature of older person's hospital to home discharge experiences. The limited range of reviews addressing the older persons gender, ethnicity, existing health conditions and reason for hospital admission is reflected in other older person research despite the international drive for inclusivity (Pitkala and Strandberg 2022). Barriers to research emanate from structural issues, for example, capacity, infrastructure and research readiness. Study design, network developments and identification of key questions need to be in place to support older person research participants (Witham et al. 2021). Older people's views on research participation vary. Some describe it as ‘an essentiality beyond one's own competence’ which they find challenging to access and understand (Haak et al. 2021, 3). However, they believed in the benefits of research for societal development to which they could contribute through their lived experience of older age (Haak et al. 2021). Adequate reporting of older persons demographic data enhances research utility, transferability and generalisability of new knowledge (Varma et al. 2021).

Older people in included studies almost universally wanted to go home. Return to the comfort and familiarity of one's own home is a natural desire for most people (Todres and Galvin 2010). This motivation to expedite discharge from hospital to home also transcends across international healthcare policy. For example, in the United Kingdom, significant investment has been made in moving healthcare closer to home, including admission avoidance and enabling early discharge (HM Government 2015). In spite of ongoing rhetoric about the need to situate people as human beings at the centre of care (Busch et al. 2019; Ellis‐Hill et al. 2022), it seems we are sometimes failing to support older people in successful hospital to home discharge. Our review highlights a need for more clarity around the older person's individual circumstances and home environment to enrich our understanding of the older person's unique discharge experience. These additional insights could support a more nuanced understanding of older people's hospital to home discharge across different contexts. In doing so, this deeper understanding might potentially illuminate some unique barriers and facilitators to a safe and effective hospital to home discharge for particular groups of older people. Through having this more detailed understanding, it may then be possible to begin to design bespoke service improvement strategies to support the hospital to home discharge for specific groups of older people.

Our review also indicates poor communication and exclusion from the discharge planning process is commonly experienced. There is a drive particularly in more developed countries to rebalance relationships between patients and HCPs often through shared decision‐making (SDM). Comprehensive reviews have investigated SDM with older people from different perspectives. A realist synthesis concludes four context‐mechanism‐outcomes need to be present to support SDM: awareness of patient and carer values, capacity to access and use care, prioritising SDM and having a person‐centred culture (Bunn et al. 2018). Programme theories likely promoting SDM are those that allow older people to feel that they are respected and understood, and that engender confidence to engage in SDM. There is a need for a ‘radial shift’ from current practice before SDM can become a reality (Bunn et al. 2018). Similar conclusions are drawn in a review of barriers (*n* = 149) and facilitators (*n* = 67) with older people with multiple chronic conditions (MCC). MCCs were perceived as a barrier to SDM although some HCPs regarded older people's experience of living with MCC as an asset. A clear offer to engage in SDM, a supportive organisational context and effective staff communication skills enhance effective SDM (Pel‐Littel et al. 2021).

A strength of this review is it has diligently followed and reported an overview of qualitative review methodology. It provides new insights into our lack of knowledge about the characteristics of the older people who have engaged in research and identified key themes regarding experiences of hospital to home discharge. These findings support recommendations to inform the design of future practice and research regarding hospital to home discharge for older people. A limitation of this review is that it is possible that not all available literature has been included as the search, although systematic, this review was limited to papers published in English. Overview of review methodology also has limitations as findings from primary studies may become further diluted. Finally, another limitation is the notable geographic bias towards reviews including primary research conducted in high‐income countries, limiting wider global extrapolation of findings.

Future research should include more detailed data regarding the older persons' individual characteristics and onward home environment. Imprecision regarding the age of participants is problematic. Although age is generally ≥ 65 years, some reporting is poor, and as longevity increases, there is an increasing need to consider the potentially different experiences and needs of older‐old adults (López et al. 2020). Data regarding gender and ethnicity is also incomplete. This misses an opportunity to enrich our understanding of the unique discharge experience for older men and women from diverse cultures and ethnic backgrounds whose roles and expectations may be quite different. Similarly, reporting of ethnicity would both identify gaps in current knowledge and enhance our understanding of the unique discharge experiences of people from varied ethnicities and cultures. Given the multi‐morbidities experienced by many older people, providing reasons for admission may be complex. However, data regarding whether admission to hospital was planned or emergency would add a further layer of understanding. More detailed reporting of demographics would move away from the homogenisation of older people and enable a more nuanced understanding of experiences in different contexts. The inclusion of the views of informal carers and/or HCPs implies these groups can offer proxy information. It is not clear why these views were included, but as our expertise in researching with older people increases (Richardson et al. 2020), the need to seek the views of others will diminish.

In clinical practice, it is important for HCPs to acknowledge that some older people's awareness of the ‘busy’ and ‘rushed’ healthcare environment as this inevitably impacts on effective communication and relationships. To enhance communication, HCPs need to be ‘in the moment’ consciously being ‘present’ (Hessel 2009) with older people to enhance collaboration and caring (Mohammadipour et al. 2017) in discharge planning. Older people should be supported to engage in SDM.

## 5 Conclusion

This overview of reviews illustrates limited research addressing the older person's gender, ethnicity, existing health conditions and reason for admission suggesting gaps in our understandings of the older person and their unique home context in existing research regarding hospital to home discharge. Healthcare practitioners can use strategies such as person‐centred care and shared decision‐making to better engage older people in discharge planning and meet their individual needs. To avoid the homogenisation and to support greater diversity and inclusivity of older people, it is suggested that future researchers in this field should provide information about the age, gender, ethnicity, health conditions, reasons for admission and the home environment. Researchers should strive to work directly with diverse groups of older people in all stages of research. This approach may provide a deeper understanding of the perspectives of older people and illuminate some unique barriers and facilitators and provide healthcare leaders, policymakers and future researchers with a more rich and nuanced understanding when planning and implementing acceptable, safe and effective hospital to home discharge for particular groups of older people.

## Author Contributions

U.K. conceived the study, designed the search methodology, screened, collected, synthesised relevant studies and supported with drafting the manuscript. F.C. and J.D. reviewed the methodology, search strategy, quality appraisal and thematic synthesis alongside supporting with the drafting and critical revision of the manuscript.

## Conflicts of Interest

The authors declare no conflicts of interest.

## Data Availability

The data that support the findings of this study are available from the corresponding author upon reasonable request.
